# Nanoindentation-induced phase transformation and structural deformation of monocrystalline germanium: a molecular dynamics simulation investigation

**DOI:** 10.1186/1556-276X-8-353

**Published:** 2013-08-15

**Authors:** Min Lai, Xiaodong Zhang, Fengzhou Fang

**Affiliations:** 1 State Key Laboratory of Precision Measuring Technology & Instruments, Centre of MicroNano Manufacturing Technology, Tianjin University, Tianjin 300072, China

**Keywords:** Monocrystalline germanium, Nanoindentation, Phase transformation, Anisotropy, Molecular dynamics simulation

## Abstract

Molecular dynamics simulations were conducted to study the nanoindentation of monocrystalline germanium. The path of phase transformation and distribution of transformed region on different crystallographic orientations were investigated. The results indicate the anisotropic behavior of monocrystalline germanium. The nanoindentation-induced phase transformation from diamond cubic structure to β-tin-Ge was found in the subsurface region beneath the tool when indented on the (010) plane, while direct amorphization was observed in the region right under the indenter when the germanium was loaded along the [101] and [111] directions. The transformed phases extend along the < 110 > slip direction of germanium. The depth and shape of the deformed layers after unloading are quite different according to the crystal orientation of the indentation plane. The study results suggest that phase transformation is the dominant mechanism of deformation of monocrystalline germanium film in nanoindentation.

## Background

Germanium plays a significant role in various fields such as solar cell, infrared optics, semiconductor, and photoelectric detection. In order to achieve nanoscale surface finishing or micro-nanometric intricate features of germanium devices, a fundamental understanding on deformation process and mechanical properties at the nanoscale becomes essential. Nanoindentation is one of the most important approaches to estimate mechanical properties in nanometer scale, which can test the modulus of elasticity, hardness, and yield stress of thin films or bulk specimens.

In recent years, many researchers have focused on phase transformations in silicon during nanoindentation by both experiments and molecular dynamics simulations. The experimental methods for characterization of phase transformation include electrical resistance test [1], Raman spectroscopy [2-6], cross-sectional transmission electron microscopy [3-5], and scanning electron microscopy [2,4,5]. Previous studies indicated that nanoindentation-induced phase transformation of monocrystalline silicon occurred, and Si-III, Si-XII, or amorphous-Si were detected after unloading [1-6]. Molecular dynamics simulation could directly observe the phase transformations of silicon and their distribution, as well as the anisotropic behavior of single crystal, during nanoindentation in detail at the atomic level. It could also be employed to study the influence of indenter shape, temperature, or other processing conditions on material deformation expediently [7-11]. Almost the same experimental methods were used to investigate the phase transformation of monocrystalline germanium in nanoindentation, and metallic β-tin phase (Ge-II) was detected under a certain pressure. It was found that the favored plastic deformation of bulk crystalline germanium in nanoindentation was caused by shear-induced twinning aligned along the {111} planes and the dislocation slip [12,13]. The explanation was that the initial plastic deformations were the twinning and dislocation slip. When the propagations of twinning and dislocation slip were blocked by increasing the load, the phase transformation started [12]. In the thin Ge film, the deformation process mentioned above was heavily influenced by the film thickness [14] and the velocity of loading [15]. At present, molecular dynamics simulation of nanoindentation of germanium is rarely found except for Zhu and Fang’s study [16]. They proposed that a pressure-induced phase transformation was the dominant deformation mechanism of the monocrystalline Ge film instead of dislocation-assisted plasticity.

In this paper, the study is focused on the surface and subsurface deformation of monocrystalline germanium during nanoindentation on the (010), (110), and (111) crystal faces, respectively. The phase transformations are shown in detail at the atomic level, and the phase transformation path as well as the deformed layers after unloading on different crystal planes was analyzed.

## Methods

### Molecular dynamics simulation method

The simulation model consists of a monocrystalline germanium workpiece and a spherical indenter. The workpiece has a size of 30 nm × 30 nm × 12 nm, including 748,461 germanium atoms. The germanium substrate includes three kinds of atoms: boundary atoms, thermostat atoms, and Newtonian atoms. The bottom outer layers of atoms in the substrate were fixed in space, and the layers neighboring them were kept at a constant temperature of 293 K to imitate heat dissipation in a real nanoindentation condition. The rigid diamond indenter was designed as a spherical shape with a radius of 10 nm and moves at a velocity of 100 m/s during loading and unloading. The maximum penetration depth was set at 5 nm, where the indenter would remain for about 2,000 time steps. Nanoindentation simulations on three different crystallographically oriented surfaces including the (010), (101), and (111) planes were conducted.

Since the Tersoff potential which considers the covalent bonds and the effect of bond angle has been used to deal with IV elements and those with a diamond lattice structure such as carbon, silicon, and germanium [16-18], and its great superiority has been shown, the interaction among the germanium atoms in this study adopts this potential. The interaction between germanium atoms and carbon atoms is depicted by a two-body Morse potential, of which the corresponding parameters were provided by Lai et al. [19].

## Results and discussion

### Identification of transformed crystal structure

Similar to monocrystalline silicon, monocrystalline germanium undergoes a complicated phase transformation during mechanical loading and unloading. Experimental investigations show that germanium would transform from its diamond cubic structure to the metallic β-tin phase when the pure hydrostatic pressure increases to about 10 GPa [20]. On fast pressure release, a metastable body-centered cubic structure with 8 atoms per unit cell (denoted BC8) [21,22] forms, while a simple tetragonal phase with 12 atoms per unit cell (ST12) [23] forms in the case of slow pressure release. The threshold pressure inducing the phase transformation mentioned above was deemed to be 12 GPa [24].

To identify the different phases of silicon and germanium formed in nanoindentation or nanocutting by molecular dynamics (MD) simulation, the coordination number is usually taken into consideration. For silicon, it is widely accepted that the atoms with coordination number 4 indicate the diamond cubic structure and the sixfold coordinated atoms are considered as the β-tin phase [7,9,11,16,25]. The atoms with coordination number 5 indicate the bct5 structure, which is considered as an intermediate in the formation of the sixfold coordinated β-tin phase [16,25] or to have some relationship with amorphous silicon or liquid-state silicon [26]. However, the way of estimating crystal phase merely according to the statistics of coordination number is not be very reliable. For example, amorphous germanium consists of 90% atoms with coordination number 4, about 10% fivefold coordinated atoms, and a small number of sixfold coordinated atoms [27], which could be easily mistaken for the mixed structure of the three phases mentioned above if the judgment criterion is just the statistic of the coordination number. Hence, in this paper, atoms with the same coordination number forming an area with the ordered structure are considered as the relevant crystal phase. The germanium atoms were colored according to their coordination number during and after nanoindentation. If atoms with the same coordination number form the ordered structure, regions with a single color would be observed. In addition, since molecular dynamics simulation can present the crystal structure in detail at the atomic level, the atomic structure of the local region was enlarged for observation to distinguish the relevant phases.

According to previous studies, the β-tin structure of germanium may undergo phase transformation into BC8-Ge or ST12-Ge on pressure release, and the transformation path depends on the rate of pressure release. Unfortunately, both BC8-Ge and ST12-Ge have the same coordination number with diamond cubic structure [24,28]. For silicon, the researchers distinguished the pressure-release-induced metastable phases from the diamond cubic structure by the characteristic that they both have a non-bonded fifth neighbor within a certain distance while diamond cubic silicon has 12 non-bonded second neighbors [7,9]. The BC8-Ge and ST12-Ge phases were transformed from the β-tin-Ge structure, which means that these two metastable phases should exist in the previous area of β-tin-Ge phase. Since molecular dynamics simulation can present the crystal structure in detail at the atomic level during nanometric machining, the approach to estimate the formation of BC8-Ge and ST12-Ge in this study is by directly observing the atoms with coordination number 4 and their crystal structure in the previous area of the β-tin-Ge phase during and after unloading.

### Phase transformation during loading

**Figure 1:**
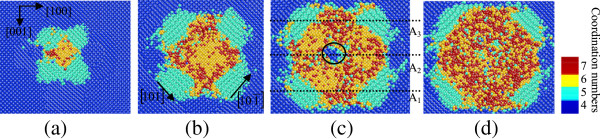
**Top cross-sectional views of phase transformed region at different depths when nanoindenting on (010) germanium surface.** At the depth of **(a)** approximately 9 nm, **(b)** approximately 7 nm, **(c)** approximately 6 nm, and **(d)** approximately 5 nm from the top of the substrate.

**Figure 2:**
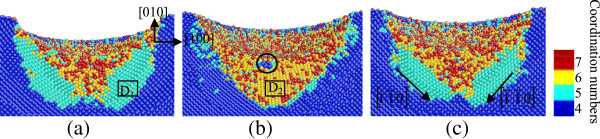
**Side cross-sectional views of phase transformed region induced by nanoindenting on the (010) germanium surface.** The surface is parallel to the (001) plane of **(a)** A_1_, **(b)** A_2_, and **(c)** A_3_ in Figure 1.

**Figure 3:**
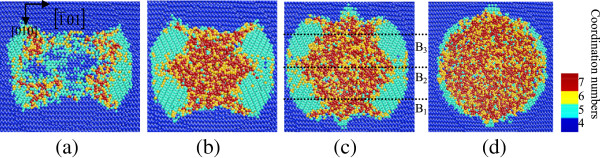
**Top cross-sectional views of phase transformed region at different depths when nanoindenting on (101) germanium surface.** At the depth of **(a)** approximately 9 nm, **(b)** approximately 7 nm, **(c)** approximately 6 nm, and **(d)** approximately 5 nm from the top of the substrate.

**Figure 4:**
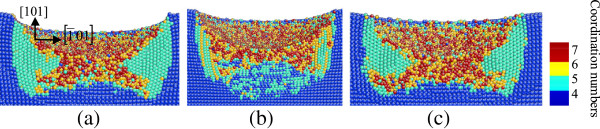
**Side cross-sectional views of phase transformed region induced by nanoindenting on the (010) germanium surface.** The surface is parallel to the (010) plane of **(a)** B_1_, **(b)** B_2_, and **(c)** B_3_ in Figure 3.

**Figure 5:**
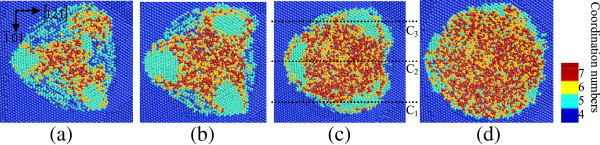
**Top cross-sectional views of phase transformed region at different depths when nanoindenting on (111) germanium surface.** At the depth of **(a)** approximately 9 nm, **(b)** approximately 7 nm, **(c)** approximately 6 nm, and **(d)** approximately 5 nm from the top of the substrate.

**Figure 6:**
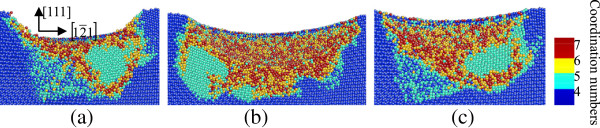
**Side cross-sectional views of phase transformed region induced by nanoindenting on the (111) germanium surface.** The surface is parallel to the $\left(\bar{1}01\right)$ plane of **(a)** C_1_, **(b)** C_2_, and **(c)** C_3_ in Figure 5.

**Figure 7:**
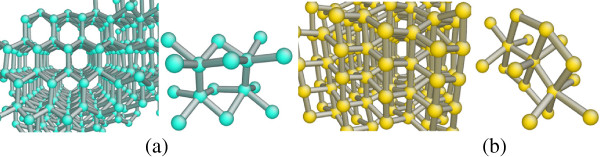
**Images of the structures formed during nanoindentation of monocrystalline germanium. (a)** bct5-Ge structure, an enlarged view of D_1_ in Figure 2a. **(b)** β-tin-Ge structure, an enlarged view of D_2_ in Figure 2b.

Figures 1 and 2 are the top cross-sectional views and side cross-sectional views of nanoindentation on the (010) germanium surface with penetration depth of 5 nm, which show the structural phase distributions at different depths from the machined surface and different sections from the side face, respectively. Figures 3 and 4 show the distributions of the transformed structure when nanoindenting on the (101) surface, while Figures 5 and 6 show those of the transformed structure nanoindented on the (111) germanium plane. The extensive crystalline structure with fivefold coordinated atoms forms around the center of phase transformed region in all cases of nanoindentation in this work. The crystal structure at the atomic level is shown in Figure 7a, which is almost the same with the structure of bct5-Si. The bct5-Si structure has a body-centered tetragonal lattice with fivefold coordinated atoms. The first-principles total-energy calculation and model potentials show that the structure is a low-energy phase of silicon and stable at ambient condition [26]. Since monocrystalline germanium is similar with silicon in many aspects such as crystal structure, physical property, and phase transformation under pressure, they always adopt the same potential in MD simulations. This crystal structure of fivefold coordinated germanium atoms is believed to be the bct5-Ge. The bct5-Ge appears around the center of the indentation region instead of being located centrally in the nanoindentations on the (010), (101), and (111) germanium surfaces, which indicates that non-hydrostatic pressure can induce transformation from diamond cubic germanium into the bct5 phase, and the same holds true for silicon [7].

It is generally accepted that monocrystalline germanium transforms from a diamond cubic structure into a β-tin structure (Ge-II) during nanoindentation. Our study indicates that the process and distribution of a structurally transformed phase are quite different when nanoindenting on various crystallographic orientation planes.

In the case of nanoindentation on the (010) plane, the phase transformation from diamond cubic structure into bct5-Ge (in cyan) occurs in the large areas surrounding the central place. The Ge-II structure (in yellow) initially appears centrally at the subsurface region beneath the indenter instead of at the region right under the tool. The atoms with coordination number 4(in black circles) shown in Figures 1c and 2b are arranged as diamond cubic structure. The stress distribution beneath a spherical indenter was obtained by a previous simulation, which shows that the maximum hydrostatic stress occurs at the surface while the maximum shear stress occurs beneath the surface during initial elastic deformation in nanoindentation with a spherical indenter [14]. In this study, the Ge-II phase initially forms at the region beneath the surface, which indicates that the hydrostatic stress is not the only determining factor for the phase transformation from diamond cubic-Ge to Ge-II, and deviatoric stress along certain directions would reduce the threshold stress triggering this phase transformation. This phenomenon is the same with that of nanoindentation on the (100) silicon surface [7]. The atomic structural details of Ge-II are shown in Figure 7b, which is an enlarged view of the region D_2_ in Figure 2b. The boundaries of different phases are mainly along the $\left[101\right],\;\left[10\bar{1}\right],\;\left[1\bar{1}0\right]\;\text{and}\;\left[\bar{1}\bar{1}0\right]$ directions, all of which belong to the same < 110 > slip direction of germanium.

When germanium was loaded in the [101] direction, the top cross-sectional views in Figure 3 show that a large area of bct5-Ge occurs around the center and the phase transformed region extends along the $\left[\bar{1}01\right]$ direction. According to the side cross-sectional views of nanoindentation on the (101) surface in Figure 4, the transformed region extends deeper in the germanium substrate in the [101] direction, and the central region under the spherical indenter presents a disordered amorphous state instead of the Ge-II phase, which occurs in nanoindentation on the (010) germanium surface. Beneath the amorphization region, a mixed structure consisting of fourfold coordinated atoms and fivefold coordinated atoms forms and extends into the substrate.

In the case of nanoindentation on the (111) germanium surface, the amorphization occurs beneath the spherical indenter, similar to that in nanoindentation on the (101) plane. Three large areas of bct5-Ge phase are arranged at 120° rotational symmetric positions around the central region with disordered atoms. Each one is surrounded by a narrow zonal region of disordered structure. Among these three regions, the mixed structure consisting of fourfold coordinated atoms and fivefold coordinated atoms exists beneath the direct amorphization region of the surface, as shown in Figures 5 and 6.

### Deformed region after unloading

**Figure 8:**
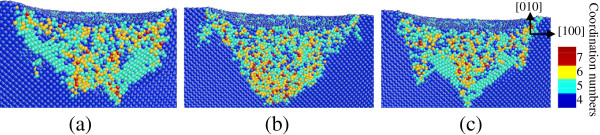
**Side cross-sectional views of the phase transformed region after unloading on the (010) germanium face.** The surface is parallel to the (001) plane of **(a)** A_1_, **(b)** A_2_, and **(c)** A_3_ in Figure 1.

Figure 8 shows the side cross-sectional views of nanoindentation on the (010) surface after unloading, corresponding to the images in Figure 2. The previous Ge-II structure has changed into a disordered amorphous structure, which generally consists of atoms with coordination numbers 4, 5, and 6. In this region, there is no crystal structure with fourfold coordinated atoms, which means that the phase transformation from Ge-II to ST12-Ge or BC8-Ge during and after unloading does not happen in our MD simulation. Instead, the Ge-II phase transforms into the amorphous structure directly. The area near the edge of the bct5-Ge region transforms into amorphous germanium while majority of those at the center retains the bct5 structure, which confirms that the bct5 structure is relatively stable in simulations [26]. It is noted that the bct5 structure is only proposed by the first-principles calculations and model potentials, and it has not been observed experimentally up to now. It is conjectured that the btc5 structure may relate to amorphous structure or liquid state [26], or is the transition state between the diamond cubic structure and β-tin phase [16,25]. The shape of the deformed layers on the (010) surface is thick at the center and thin near the edge after unloading. The boundary of diamond structure and transformed phase is still parallel to the $\left[1\bar{1}0\right]\;\text{and}\;\left[\bar{1}\bar{1}0\right]$ directions, respectively.

**Figure 9:**
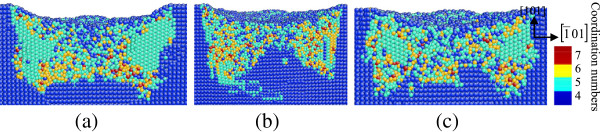
**Side cross-sectional views of the phase transformed region after unloading on the (101) germanium face.** The surface is parallel to the (010) plane of **(a)** B_1_, **(b)** B_2_, and **(c)** B_3_ in Figure 3.

When the indentation plane is the (101) surface, amorphous structures were observed in the central region and most of the surrounding bct5 phase still exists after unloading, as shown in Figure 9. The majority of the mixed structures consisting of fourfold and fivefold coordinated atoms were restored to initial diamond cubic structure, which causes the thickness of the deformed layers near the edge of the transformed region to be greater than that of the center area on the (101) surface. Moreover, the boundary of the transformed region is along the [101] direction.

**Figure 10:**
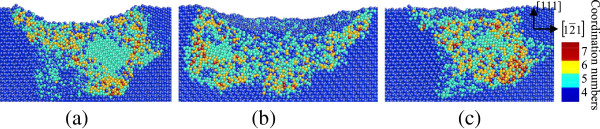
**Side cross-sectional views of the phase transformed region after unloading on the (111) germanium face.** The surface is parallel to the $\left(\bar{1}01\right)$ plane of **(a)** C_1_, **(b)** C_2_, and **(c)** C_3_  in Figure 5.

In the case of nanoindentation on the (111) germanium plane, most of the mixed structures formed during loading were restored to diamond structure during and after unloading, and most of the bct5-Ge structures still exist (Figure 10). Another region of the transformed phase assumes a disordered amorphous state.

## Discussion

The results of the MD simulations above indicate that the phase transformation path and distribution of monocrystalline germanium during nanoindentation are different according to the crystallographic orientation of the loaded crystal plane. Monocrystalline germanium has a diamond-like structure, which follows the face-centered cubic (fcc) Bravais lattice. The lattice consists of two basis atoms and can be considered as two inter-penetrating fcc lattice, one displaced about 1/4 of the body diagonal from the other along the [111] direction. According to the crystal structure, the atomic arrangement on the (010) plane of germanium has a fourfold rotational symmetry, the (111) plane has a threefold rotational symmetry, and the (101) plane has two different twofold rotational symmetric directions. In this study, the top cross-sectional views of the (010), (101), and (111) crystal planes show that the symmetrical characteristic of transformed phase distribution has a high degree of consistency with the symmetry of the indented plane itself. Since a spherical indenter was used in the simulation, the effects of asymmetrical stress induced by the indenter shape can be avoided.

**Figure 11:**
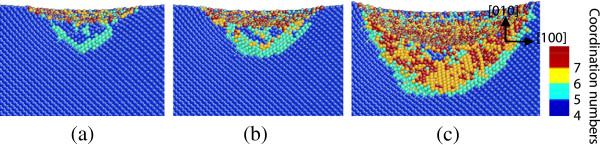
**The process of phase transformation in nanoindentation on the (010) germanium surface.** The indentation depth is **(a)** approximately 1.2 nm, **(b)** approximately 2 nm, and **(c)** approximately 4.5 nm.

During loading, the diamond cubic germanium under the spherical indenter transforms into Ge-II phase when nanoindenting on the (010) surface, while direct amorphization occurs beneath the tool in the cases of nanoindentation on the (101) and (111) surface. On unloading, the Ge-II phase on the subsurface of the (010) plane transforms into amorphous state. Some experiments reported that germanium film could transform into metallic Ge-II phase under load and then into amorphous germanium during unloading, or else it could directly transform from diamond cubic structure to amorphous germanium [13,14]. The path of phase transformation has something to do with sample preparation and loading condition. This study results in understanding both the phase transformation path and distributions in germanium, proving that the crystalline orientation also influences the path of phase transformation in nanoindentation of germanium. Figure 11 presents the process of phase transition in nanoindentation on the (010) plane. The bct5-Ge initially appearing under the indenter transforms into Ge-II with continuing loading, which indicates that the bct5-Ge could be an intermediate in the formation of Ge-II phase similar to silicon, as mentioned in previous researches [16,25]. However, the bct5-Ge in the surrounding area does not transform into Ge-II with continuing loading. In addition, the bct5-Ge forming in nanoindentation on the (101) and (111) planes does not transform into Ge-II structure either. These phenomena suggest that pressure with specific directions could induce phase transition from bct5-Ge to Ge-II structure. In other words, axial force with specific directions could trigger phase transformation from diamond cubic germanium to Ge-II phase besides the hydrostatic stress.

The bct5-Ge structure always forms around the center of the transformed region and almost still exists after unloading. At the same time, the majority of the mixed structure with fourfold and fivefold coordinated atoms forming under pressure stress recovers the diamond structure after load relief. The calculated stress in this region is about 6 GPa, which is much lower than the threshold stress initiating the phase transformation. Hence, it is suggested that the mixed structure mentioned previously is the distorted diamond cubic structure. The elastic deformation of this region arises on loading, and it returns back to the original diamond structure during unloading. The change in the coordination number of the atoms may comes from the inappropriate cutoff radius for calculation of the nearest neighbors.

The borders of the transformed regions are mostly parallel to germanium’s slip direction of < 110 >, which influences the shape of deformed layers after nanoindentation. The maximum extending depth of the deformed layers also differs based on the crystal orientation of the germanium contact surface. The distribution of deformed layers on the (111) germanium surface is more compact and has the thinnest depth from the contact surface into the substrate, while those on the (010) and (101) surfaces have great difference in depth on various regions and extend deeper into the substrate.

The recovery of the central location in nanoindentation on unloading is recorded in Table 1. It is noted that the recovery mentioned here is the combination of the elastic recovery of the distorted diamond cubic structure of Ge and the volume change induced by the phase transformation during unloading. In the case of nanoindentation on the (010) plane, Ge-II at the central location transforms into amorphous germanium on unloading, which is < 20% less dense than Ge-II [13,29], and mainly accounts for the expressional recovery. The central surface of the (010) and (111) planes presents amorphous state on loading and after unloading. However, the loading amorphous structure is different in coordination numbers from the unloading amorphous state. The latter is more similar with the amorphous germanium in normal condition [27,29]. Theoretical investigation using the Tersoff potential showed that a gradual low-density to high-density amorphous transformation occurred [29], and the high-density amorphous phase is similar to liquid Ge. Hence, besides the elastic recovery from the distorted diamond cubic structure of germanium, the recoveries of the indentation on the (101) and (111) face on unloading are either from the phase transformation from high-density amorphous phase to low-density amorphous Ge, or else from the elastic recovery of distorted amorphous germanium on stress relief, which depends on the stress in the amorphous region during loading, since the nature of recovery on the (010) plane is variant from that on the (101) and (111) planes on unloading, as analyzed above. Moreover, the central deformed layer on the (010) plane is much deeper than that on the (101) and (111) planes. As a result, the recovery on the (010) surface of germanium is bigger than that on the (101) and (111) planes on unloading. The conditions of deformed layers on different crystallographic orientation surfaces are listed in Table 1.

**Table 1:** Conditions of deformed layers on unloading

	**Crystallographic orientation**		
	**(010)**	**(101)**	**(111)**
Maximum depth of deformed layers (nm)	9.1	9.0	5.8
Recovery of the center (nm)	3.7	3.0	2.8
Description of deformed layers	Thin at the center and thick at the circumference	Thick at the center and thin at circumference	Relatively uniform thickness

## Conclusions

This study presents the nanoindentation-induced phase transformation and deformation of monocrystalline germanium at the atomic level. The path of phase transformation and distribution of the transformed region on different crystallographic orientations of the loaded planes were investigated, which obviously indicate the anisotropy of the monocrystalline germanium. The conclusions obtained are as follows:

(1) The large area of phase transformation from diamond cubic structure to Ge-II phase was observed in nanoindentation on the (010) germanium surface in the subsurface region beneath the spherical indenter, while the transformation of direct amorphization occurs when nanoindenting on the (101) and (111) germanium surfaces.

(2) The phase transformation and direct amorphization are the primary mechanisms of plastic deformation for monocrystalline germanium film in nanoindentation instead of the formation and multiplication of dislocations. These transformed phases mostly extend along the < 110 > slip direction of germanium.

(3) The thinnest depth of deformed layers after unloading was obtained in nanoindentation on the (111) germanium surface, and the depth distribution is also more compact than that of the other two surfaces from the side cross-sectional views after indentation. The recovery of nanoindentation on the (010) germanium plane is greater than that on the (101) and (111) planes.

## Competing interests

The authors declare that they have no competing interests.

## Authors’ contributions

FZF conceived of the research work and participated in the analyses. XDZ participated in its design, coordination, and analyses. ML carried out the molecular dynamics simulations of nanoindentation on monocrystalline germanium, analyzed the simulation results, and drafted the manuscript. All authors read and approved the final manuscript.
